# Meniscal extrusion and degeneration during the course of osteoarthritis in the Murine collagenase‐induced osteoarthritis model

**DOI:** 10.1002/jor.23909

**Published:** 2018-04-25

**Authors:** Lizette Utomo, Susanne M. Eijgenraam, Duncan E. Meuffels, Sita M. A. Bierma‐Zeinstra, Yvonne M. Bastiaansen‐Jenniskens, Gerjo J. V. M. van Osch

**Affiliations:** ^1^ Department of Orthopaedic Surgery, Erasmus MC University Medical Center Rotterdam Rotterdam The Netherlands; ^2^ Department of Radiology and Nuclear Medicine, Erasmus MC University Medical Center Rotterdam Rotterdam The Netherlands; ^3^ Department of General Practice, Erasmus MC University Medical Center Rotterdam Rotterdam The Netherlands; ^4^ Department of Otorhinolaryngology, Erasmus MC University Medical Center Rotterdam Wytemaweg 80, Room Ee 16.55, 3015 CN Rotterdam The Netherlands

**Keywords:** meniscus, meniscal degeneration, meniscal extrusion, osteoarthritis, CIOA

## Abstract

Meniscal damage is, despite its major role in knee osteoarthritis (OA), often neglected in OA animal models. We evaluated structural meniscal degeneration during the course of OA in the murine collagenase‐induced OA (CIOA) model. To investigate this, OA was induced in the knee joints of 33 male C57BL/6 mice by an intra‐articular injection of 10U collagenase. The mice were sacrificed after 1, 3, 7, 14, 28, and 56 days, and the knees were harvested and processed for histological analysis. As control, six knees were obtained from 16‐week‐old mice in which no OA was induced. Meniscal damage, meniscal extrusion, and articular cartilage damage were evaluated on thionin‐stained sections. Associations between parameters of interest were evaluated with Spearman rho correlation tests. When compared to non‐OA knees, meniscal extrusion was visible from day 1 onwards and meniscal degeneration had a tendency to increase over time. The meniscus damage appeared around the same time as articular cartilage damage (day 14–28) and was statistically significantly more pronounced anterior than posterior, and no differences were seen between medial and lateral menisci. Meniscus and articular cartilage damage were moderately associated in the CIOA knees (*ρ* = 0.57; 95%CI [0.23–0.78]). Our findings suggest that the CIOA model is a valuable model to study the role of meniscal damage during OA progression and can support the development of future preventative treatment strategies. © 2018 The Authors. *Journal of Orthopaedic Research*® Published by Wiley Periodicals, Inc. on behalf of the Orthopaedic Research Society. J Orthop Res 36:2416–2420, 2018.

Meniscal degradation is, in spite of its critical role in knee osteoarthritis (OA), often neglected in OA animal models. Meniscal damage is one of the strongest identified risk factors for the development and progression of knee OA.^1^, ^2^, ^3^, ^4^, ^5^, ^6^, ^7^ In addition, indications that meniscal extrusion, that is, radial displacement of the meniscus outside of the joint cartilage margin, is independently related to knee OA development have been reported previously.^8^, ^9^, ^10^ Meniscal integrity is, therefore, an important factor in the long‐term health of the knee joint.^11^ Paradoxically, little is known of the exact relationship between meniscal degradation and cartilage degeneration in the development of knee OA.

Murine models for OA are frequently used to study the etiopathogenesis of knee OA in fundamental and translational studies, due to the possibility to study the disease on a pathophysiological level, or to study the effects of an experimental therapy.^12^ Despite its major role in knee OA, the menisci are grossly neglected in the diagnosis of murine knee OA. A frequently used enzyme‐based model is the collagenase‐induced OA (CIOA) model,^13^ where highly purified collagenase is injected intra‐articularly and affects joint ligaments, resulting in joint instability.^13^, ^14^ Another often used murine OA model is the surgical destabilization of the medial meniscus (DMM),^15^ a model in which the ligament that attaches the medial meniscus to the tibia is transected, resulting in an instable and displaced meniscus. Recently, a systemic evaluation method for degeneration of the meniscus in experimental OA was established by Kwok et al.^16^ In the study, the authors reported insights on the structural changes of the menisci during aging and OA and have shown that in the DMM model, meniscal damage and articular cartilage damage develop synchronously from day 14 onwards. No studies have been conducted on the elapsed meniscal degeneration in the CIOA model. Therefore, the aim of this study was to evaluate meniscal damage during the course of experimental knee OA in the CIOA mouse model, immediately after OA onset.

## METHODS

### Induction of Experimental OA

The animal experiments were carried out in correspondence with the ARRIVE Guidelines for Reporting Animal Research,^17^ and with the approval of the Animal Ethical Committee of the Erasmus Medical Center (approval no. EMC 3246 (114‐14‐01)). OA was induced using the CIOA model in the right knees of 33 male C57BL/6J01aHsd mice (28.4 ± 3.1 g; 12–14 weeks old; Envigo, Cambridgeshire, UK) as described previously.^13^ Briefly, the mice were randomly taken from their cages and were anesthetized with 3% isoflurane/0.8 L O_2_/min (Pharmachemie BV, Haarlem, the Netherlands). The knees were sprayed with 70% ethanol (BoomLab, Meppel, the Netherlands). A dermal incision was then made at the height of the patellar tendon and a 6 μl solution containing 10 U collagenase from *Clostridium histolyticum* (Sigma–Aldrich, St. Louis, MO) in saline (Sigma–Aldrich) was injected intra‐articularly in the right knees. All animals were housed at the Experimental Animal Facility of the Erasmus Medical Center in standard caging under a standard 12‐h light/dark cycle in groups of 3–9 including cage enrichment and received acid tap water and standard chow ad libitum. The mice were sacrificed by cervical dislocation 1, 3, 7, 14, 28, or 56 days after CIOA induction and the knees were processed for histological analysis. The final number of knees used for analysis was: 8 mice at day 1, 7 mice at day 3, 3 mice at day 7, 9 mice at day 14, 3 mice at day 28, and 3 mice at day 56. As controls, six naïve knees were obtained from three 16‐week‐old mice in which no OA was induced.

### Histological Scoring of Structural Meniscal Damage, Meniscal Extrusion, and Articular Cartilage Damage

The knees were harvested and fixed in 4% formaldehyde (BoomLab) for 10 days and decalcified for 10 days in 10% ethylenediaminetetraacetic acid (Sigma–Aldrich). The tissue was then dehydrated in an ascending series of alcohol, embedded in paraffin, and sectioned serially at 6 μm in the coronal plane. The sections were stained with thionin (Sigma–Aldrich) and images were taken with a NanoZoomer 2.0‐HT slide scanner (Hamamatsu, Hamamatsu City, Japan).

Meniscal damage was assessed according to the validated method described by Kwok et al.^16^ in which the menisci were evaluated based on surface integrity, cellularity, and staining intensity, with a maximum possible score of 21. The scoring was separately conducted by two researchers experienced in histological grading (LU and SME) in a complete observation‐blinded manner, meaning unaware of time‐point, case‐control status, and each other's scores. The inter‐observer reliability of the meniscus damage scoring was excellent, with an ICC of 0.84 (95%CI [0.63–0.93]).

Meniscal extrusion of the medial and lateral meniscal body was assessed on the same sections as used for histological evaluation. Extrusion is where the meniscus is partially or totally displaced from the tibial cartilage surface.^9^ This feature was scored from 0 to 4 where 0 = no extrusion, 1 = mild extrusion, 2 = moderate extrusion, 3 = severe extrusion, 4 = complete displacement of the meniscus. The assessment for meniscal extrusion was performed by LU and its evaluation extensively discussed with the co‐authors (SME, DM, GJVMvO, and YMBJ).

Structural articular cartilage damage was assessed in all four quadrants of the knee in four sections according to a modified grading and staging score for murine cartilage that was initially based on the score described by Pritzker et al.^18^ The score was determined by multiplying a grade (0–6) and a stage (0–4) and the maximum score of four sections of each quadrant was evaluated, accounting for a total of 16 scores throughout the entire knee joint. The score of the four quadrants was then summed to determine the total articular cartilage damage score in the knee resulting in a maximal possible score of 96. The ICC of the cartilage score was 0.81 (95%CI [0.42–0.84]).

### Statistics

Calculations for the histological scores were conducted with MS Excel 2016 (Microsoft, Redmond, WA) and IBM SPSS 23.0 (IBM, New York, NY) was used for statistical evaluation. Mann–Whitney *U* tests were conducted to evaluate statistically significant differences of the non‐parametric values of interests (i.e., meniscus damage and meniscal extrusion) between independent groups (i.e., per time point compared to the non‐OA knees). The association between meniscus damage and articular cartilage damage was evaluated with a Spearman rho correlation test followed by Bonferroni correction and bootstrap‐based calculations to calculate the 95% confidence interval (95%CI). *p*‐Values of <0.05 were considered statistically significant.

## RESULTS

### Meniscus Extrusion and Damage During the Course of Experimental OA

**Figure 1:**
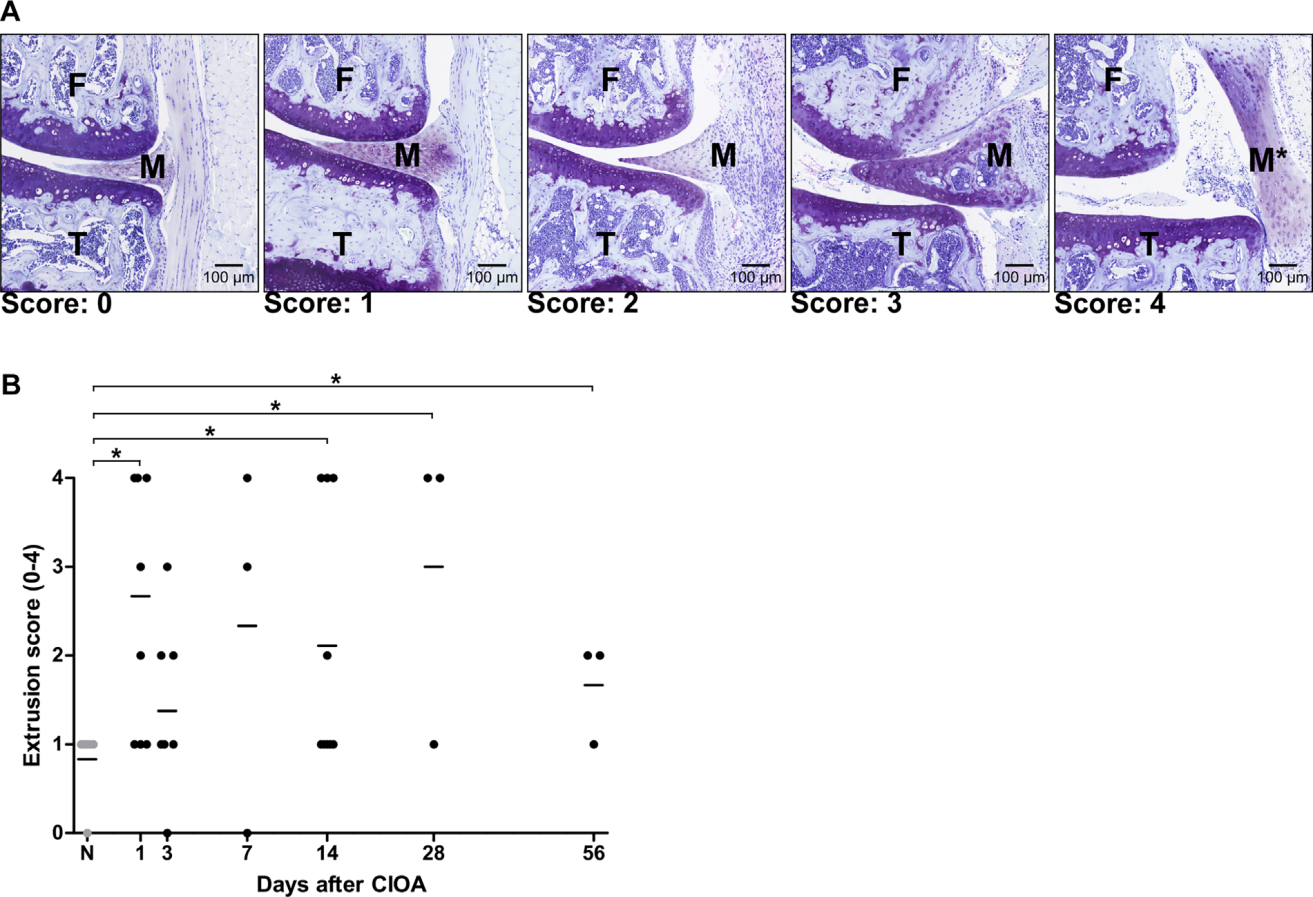
Meniscus extrusion during OA. (A) Representative images of five degrees of meniscal extrusion on thionin‐stained sections. F, femur condyle; T, tibia; M, meniscus, M*, displaced meniscus. (B) Meniscal extrusion score after induction of OA with 0 = no extrusion, 1 = mild extrusion, 2 = moderate extrusion, 3 = severe extrusion, 4 = complete displacement of the meniscus. Each symbol represents a data point of the individual knees and the horizontal lines represent the median value. *p < 0.05.

**Figure 2:**
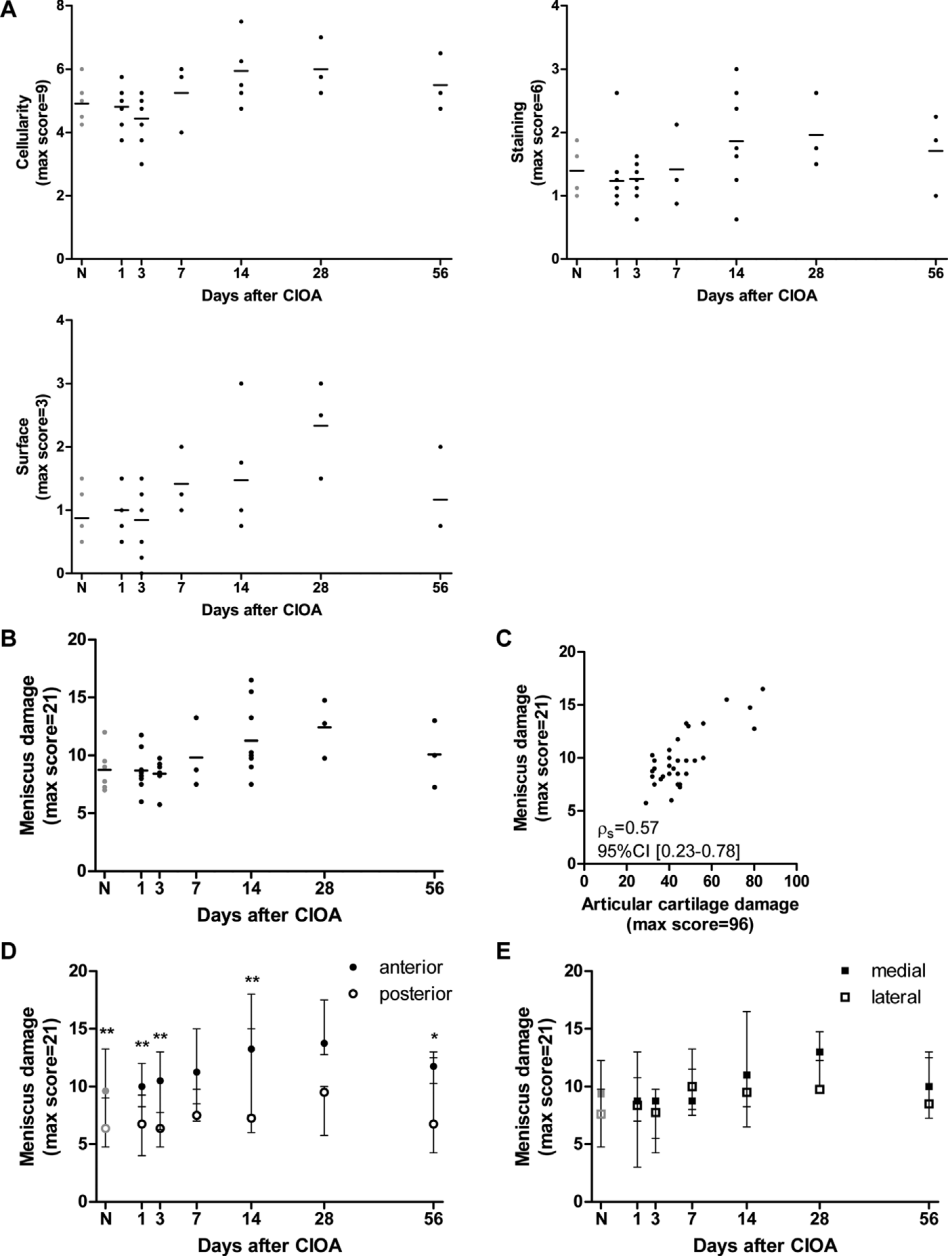
Meniscus damage during the course of collagenase‐induced OA in mice knees. (A) Subdomains of meniscus damage score; cellularity, staining intensity, surface integrity. Each symbol represents a data point of the individual knees and the horizontal lines represent the median value (B) Plot showing that meniscus damage was mild up until 56 days after induction of OA. Each symbol represents a data point of the individual knees and the horizontal lines represent the median value. (C) Spearman correlation between meniscus damage and articular cartilage damage in the CIOA knees. Differences in meniscus damage between (D) anterior and posterior, and (E) medial and lateral sides of the knees. The data are show as median with whiskers from minimum to maximum. *p < 0.05; **p < 0.01.

Meniscal extrusion was visible (Fig. 1A) and statistically significantly more present than in the non‐OA knees from day 1 onwards (Fig. 1B). No differences in extrusion were seen between the medial and lateral sides (data not shown). Meniscal degeneration was evaluated for surface structure, cellularity, and matrix staining and all three parameters had a tendency to increase over time compared to the non‐OA knees from day 14 on, albeit not significantly (Fig. 2A). When the three parameters were combined, the total meniscus damage score in the CIOA knees tended to be higher at day 14 and 28 than in the non‐OA knees (Fig. 2B). Meniscus and articular cartilage damage were moderately associated (*ρ* = 0.57; 95%CI [0.23–0.78]; Fig. 2C) in the CIOA knees and the meniscus damage appeared around the same time as articular cartilage damage (day 14‐28; data not shown). As for the locations within the knees where the damage appeared, meniscus degeneration was more pronounced anterior than posterior in both the CIOA knees and non‐OA knees (Fig. 2D). No differences were seen between medial and lateral menisci (Fig. 2E).

## DISCUSSION

We assessed meniscal extrusion and degeneration during the course of OA in the murine CIOA model. The results of this study suggest that structural meniscal degeneration appears simultaneously with articular cartilage degeneration and that they are correlated, indicating that meniscal degeneration is an important parameter when assessing OA in the CIOA model. Despite the fact that the major role of the meniscus in the development of knee OA is well established,^1^, ^3^, ^4^, ^5^, ^6^, ^7^, ^8^, ^9^, ^10^ this is the first study evaluating meniscal damage in the murine CIOA model. In another study by Kwok et al., meniscal degeneration was assessed in the DMM model.^16^ As meniscal damage was evaluated only 14 days after OA induction, relevant information during early OA onset may have been missed. In our study, we have found that meniscal damage and cartilage damage appear simultaneously, which is in concordance with findings by Kwok et al.^16^ We have additionally shown that meniscal extrusion was higher after one day in the collagenase‐injected knees than in the non‐OA knees. These findings suggest that the injected collagenase might have also affected the meniscal ligaments that, due to mechanical loading might have become insufficient, leading to meniscus extrusion. The degenerating processes of joint tissues in early stage knee OA is not limited to articular surface cartilage, but also affects meniscus integrity, as suggested in previous literature.^6^ Our findings may lead to a deeper understanding of the cascade of the development of knee OA and the complex interplay and role of the meniscus in this context. Ultimately, these insights may contribute to the development of effective therapeutic options for early OA.

Although OA animal models are useful tools to study diseases, they have limitations as well. The CIOA model that is used in this study can be categorized as a classic model for immediate joint instability and critical structural damage.^14^, ^19^, ^20^ Even though the CIOA mouse is a widely used model for knee OA, as it presents with OA characteristics such as structural cartilage damage, osteophytes, synovitis, and joint instability, there are obvious important differences between murine and human menisci. The meniscus morphology in mice differs from human menisci; mice menisci are thicker and less symmetrical in the proximal–distal direction because of the posture of the animal, since a mouse knee is in a more flexed position than a human knee. Moreover, there are differences in histological staining profiles. The staining intensity increases with age and degree of degeneration in human menisci, whereas in mice this is reversed as the staining intensity appears less intense and is disrupted in older subjects.^16^, ^21^ The reason for these differences and the meaning for the process of OA development is not clear and must be taken in consideration when assessing meniscal damage in a murine OA model.

## CONCLUSION

To conclude, several studies have shown a correlation between extrusion of the meniscal body and knee OA^8^, ^9^ and meniscal extrusion is known to be independently related to cartilage loss.^8^, ^9^, ^10^, ^22^ The generally accepted hypothesis is that an extruded meniscus modifies the load distribution and weight‐bearing abilities within the knee joint, eventually resulting in the development of knee OA.^8^, ^22^ Our study shows that meniscal extrusion appears early in the CIOA mouse model, and that meniscal damage and articular cartilage damage occur simultaneously. This highlights the CIOA model as a valuable model to study the role of meniscal damage during OA progression and the development of future preventative treatment strategies.

## AUTHORS' CONTRIBUTIONS

LU performed the animal experiments, processed samples, analyzed and interpreted the data, and wrote the manuscript. SME analyzed and interpreted the data, and wrote the manuscript. DEM interpreted the data and edited the manuscript. SMAB‐Z edited the manuscript. YMBJ and GJVMvO guided the animal experiment, interpreted the data, and edited the manuscript. All authors approved the final version of the manuscript.

## ETHICAL APPROVAL

The animal experiments were carried out with the approval of the Animal Ethical Committee of the Erasmus Medical Center, approval no. EMC 3246 (114‐14‐01).
